# Induction of cytopathic effect and cytokines in coxsackievirus B3-infected murine astrocytes

**DOI:** 10.1186/1743-422X-10-157

**Published:** 2013-05-21

**Authors:** Jun Zeng, Gefei Wang, Weizhong Li, Dangui Zhang, Xiaoxuan Chen, Gang Xin, Zhiwu Jiang, Kangsheng Li

**Affiliations:** 1 Key laboratory of infectious diseases and molecular immunopathology of Guangdong province, Department of Microbiology & Immunology, Shantou University Medical College, 22 Xinling Road, Shantou 515041, P.R. China; 2 Research Center of Translational Medicine, Second Affiliated Hospital of Shantou University Medical College, Shantou 515065, P.R. China

**Keywords:** Viral infection, Coxsackievirus, Astrocyte, Cell death, Cytokine, Neuroinflammation

## Abstract

**Background:**

Coxsackievirus commonly infects children and occasionally causes severe meningitis and/or encephalitis in the newborn. The underlying mechanism(s) behind the central nervous system pathology is poorly defined.

**Methods:**

It is hypothesized that astrocytes may be involved in inflammatory response induced by CVB3 infection. Here we discuss this hypothesis in the context of CVB3 infection and associated inflammatory response in primary mouse astrocytes.

**Results:**

The results showed that coxsackievirus receptor (CAR) was distributed homogeneously on the astrocytes, and that CVB3 could infect and replicate in astrocytes, with release of infectious virus particles. CVB3 induced cytopathic effect and production of proinflammatory cytokines IL-1β, TNF-α, IL-6, and chemokine CXCL10 from astrocytes.

**Conclusion:**

These data suggest that direct astrocyte damage and cytokines induction could be a mechanism of virus-induced meningitis and/or encephalitis.

## Background

Coxsackieviruses are important human pathogens. Although most coxsackievirus infections are mild, serious complications such as meningitis, paralysis and myocarditis are not rare. Coxsackievirus B3 (CVB3) is the most frequently involved in human myocarditis. Recently, increasing cases of CVB3-associated meningitis have been reported [1,2]. Neuroinflammation and neuronal loss have been shown in the central nervous system (CNS) infected by CVB3. Using a neonatal mouse model of CVB3 infection, a previous study has shown that neuronal precursor cells were preferentially targeted and ensuing neuronal apoptosis [3]. Microglia and astrocytes are the major immune cells of the CNS. Our preliminary study showed that microglia were spared from CVB3 infection (unpublished data), whereas the role of astrocytes in CVB3 infection and/or its pathogenesis has not been studied.

Expression of specific receptors is often an important determinant of a cell’s susceptibility to infection. Coxsackievirus and adenovirus receptor (CAR) is used by coxsackievirus to attach cell surface [4]. CAR is a part of the tight junction in blood–brain barrier (BBB) and appears to function in cell adhesion or intercellular recognition and participate in neural migration in the CNS [5,6]. Viral meningitis is frequently associated with BBB disruption, thus enabling the entry of virus and inflammatory cells into the CNS [7]. BBB is formed by endothelial cells, surrounded by basal lamina and astrocytes endfeet. As our preliminary study showed that CAR was expressed on murine astrocytes, we hypothesized that CVB3 infection process in the CNS involves astrocytes.

Damage to the CNS tissues may result from viral replication or as a consequence of inflammatory response [8]. Cytokines are important mediators of the inflammatory response to many stimuli, including viral infections. Cytokines such as IL-1β, TNF-α and IL-6 enhance the permeability of the BBB both in vitro [9,10] and in vivo [11]. Therefore, in this study, we have examined the CVB3 susceptibility of astrocytes and the response of inflammation cytokines (IL-1β, TNF-α, IL-6) and chemokine CXCL10 by using primary murine astrocytes.

## Results

### Isolation of murine primary astrocytes and detection of Coxsackievirus and Adenovirus receptor (CAR)

**Figure 1:**
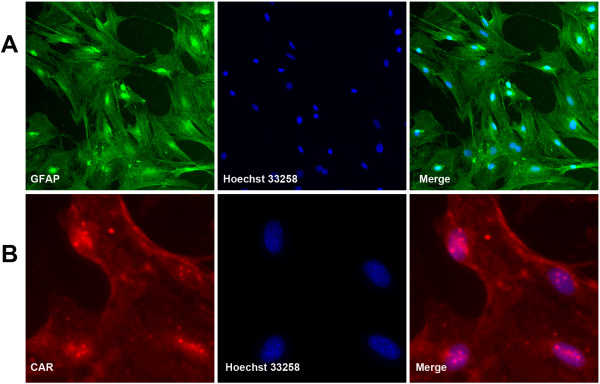
**Expression of Coxsackievirus receptor (CAR) on primary astrocytes.** Cells were treated as described in the text, fixed with Paraformaldehyde, permeabilized and stained with specific antibody. (**A**) A highly homogeneous population of astrocyte monolayers stained with anti-GFAP antibody (a marker for astrocytes) and Alexa Fluor 488 goat anti-rabbit secondary antibody (magnification 200×). (**B**) Homogeneous distribution of CAR as shown by fluorescence using anti-CAR (a marker for Coxsackievirus receptor) and Cy3-conjugated rabbit anti-mouse secondary antibody. The nuclei were counterstained with Hoechst 33258 (magnification 400×).

The purity of isolated astrocytes from neonatal mice was >95%, which was the average number of the GFAP-positive cells calculated from 3 independent experiments by observing 10 random fluorescence fields (Figure 1A). CAR was distributed homogeneously on the astrocytes (Figure 1B).

### Astrocyte susceptibility and replication of CVB3

**Figure 2:**
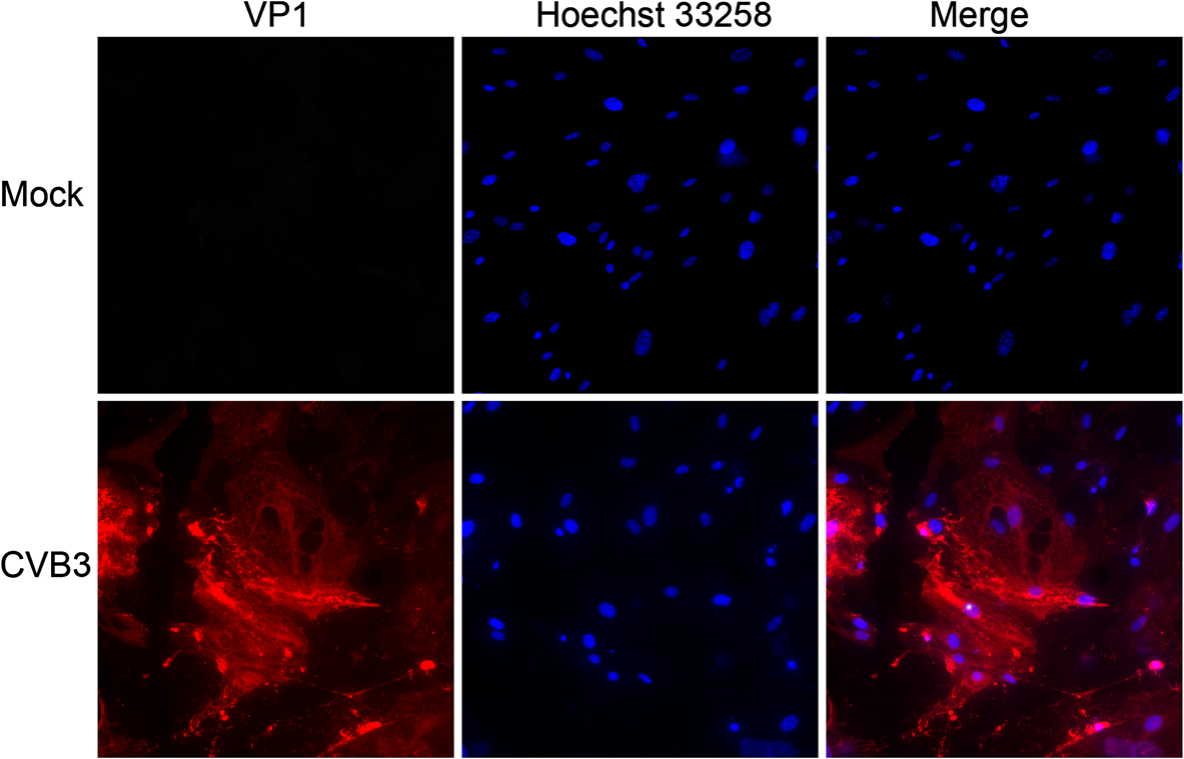
**Intracellular distribution of viral VP1 protein in CVB3-infected astrocytes at 24 hpi.** Astrocytes were either exposed to CVB3 at an MOI of 2 or to virus-free preparations (Mock). After 24 h of incubation, cells were treated as described in the text, and CVB3 particles were examined by indirect immunofluorecence with anti-VP1 antibody and Cy3-conjugated rabbit anti-mouse secondary antibody (magnification 200×).

The presence of CAR on astrocytes suggested that CVB3 could infect astrocytes. To confirm this, an indirect immunofluorescence assay was performed to observe virus infection. Astrocytes displayed a maximal virus capsid protein (VP1) expression at 24 hpi, while no astrocytes in the mock group were positive for VP1 (Figure 2).

**Figure 3:**
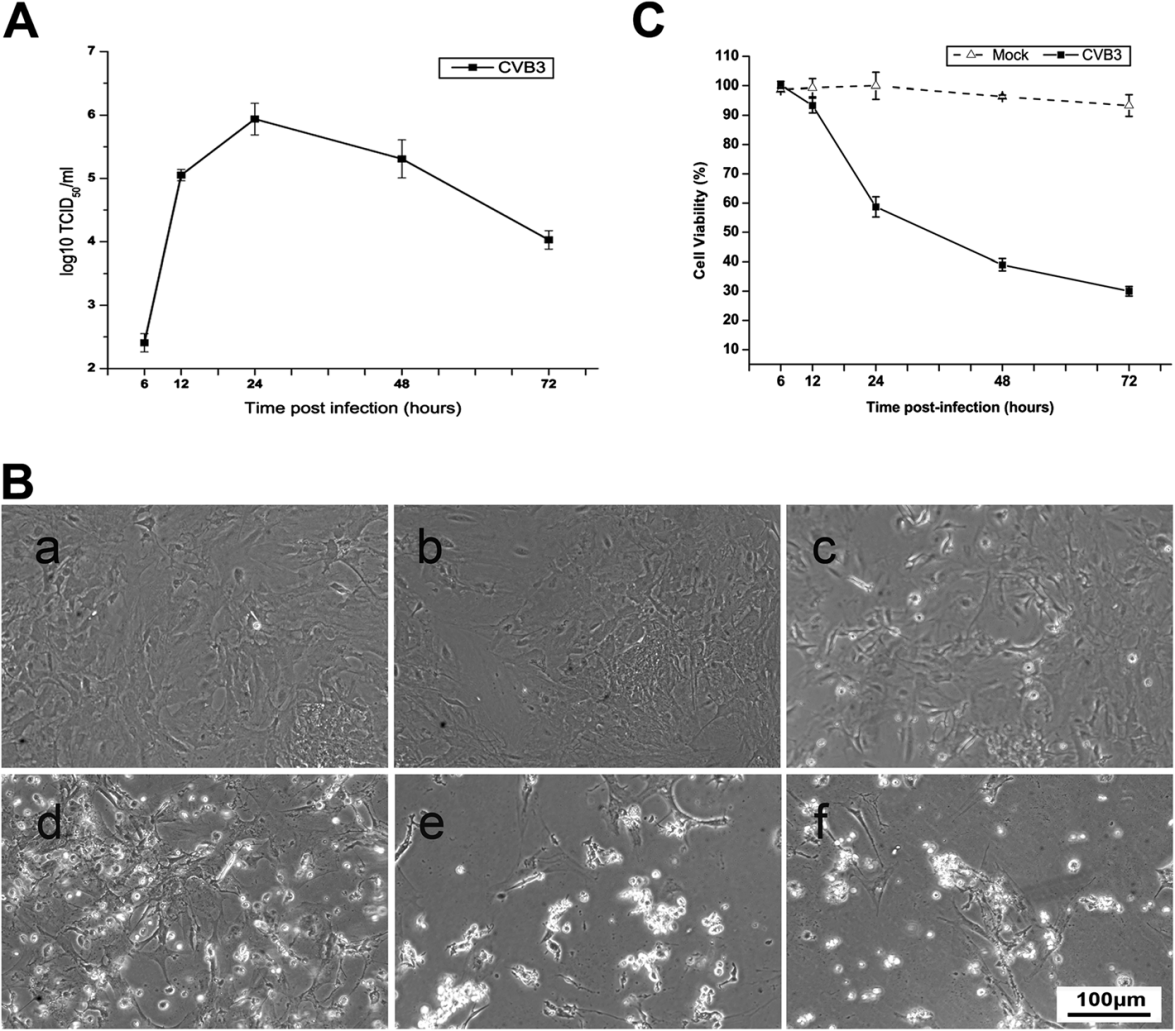
**CVB3 replication and induction of cytopathic effect (CPE) in astrocytes.** (**A**) Viral growth curve of CVB3. The culture supernatants were collected at the indicated time points and the production of infective virus particles was determined using TCID_50_ assay on Vero cells. (**B**) CVB3-induced cytotoxicity in astrocytes. Photographs were taken by light microscopy at 6, 12, 24, 48, and 72 hpi by CVB3 (**b-f**) and mock infection (**a**). CPE was observable from 24 hpi. (**C**) Viability of astrocytes was assessed by the CCK-8 kit. Values represent the mean ± SD of astrocytes from three independent experiments.

To examine whether CVB3 can produce progeny virus after infection, the cultures were exposed to CVB3 at an MOI of 0.1. Viral growth curve in Figure 3 shows that CVB3 yields increased gradually with time and peaked at about 10^6^ TCID_50_ at 24 hpi, with slow descent thereafter due likely to cell death.

### CVB3 infection induced cytopathic effect (CPE) in astrocytes

From 12 hpi, CVB3-mediated CPE was monitored microscopically. While the mock-infected cells had round phase-bright cell bodies with many neurites and smooth contours, the infected cells showed distinct morphological alterations including rounding up, appearance of granules, cytoplasmic blebbing, and detachment from the monolayer. More cells died as time went by (Figure 3B). The degree of cellular damage was estimated using the cell counting kit-8, which measures the cytoplasmic dehydrogenase activity in viable cells. We observed significant loss of cell viability by 24 hpi (58.7±3.4% viable), 48 hpi (39.0±2.1%) and 72 hpi (30.2±1.6%) (Figure 3C).

### CVB3 induced proinflammatory cytokines IL-1β, TNF-α, IL-6 and chemokine CXCL10 expression both at mRNA and protein levels

**Figure 4:**
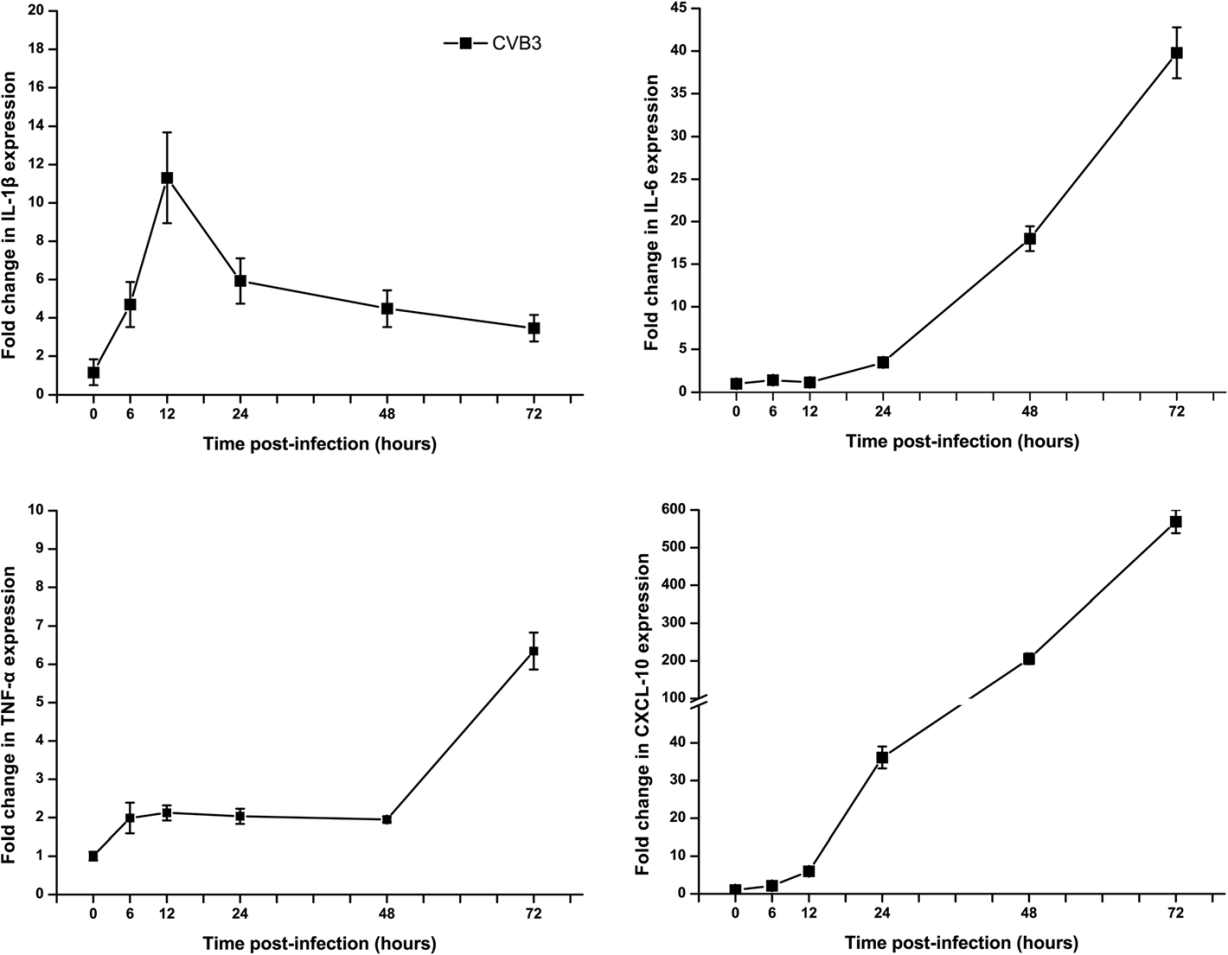
**Cytokines and chemokine mRNA levels in CVB3-infected astrocyte as measured by Real time-PCR.** The levels of expression were normalized against β-actin. The y-axis depicts the fold expression relative to mock infection.

Proinflammatory cytokine response in the CVB3-infected astrocytes was investigated by real-time PCR assay of four major cytokines IL-1β, TNF-α, IL-6, and CXCL10. A time-dependent up-regulation of their mRNA expression by CVB3 can be seen in Figure 4. IL-1β and TNF-α were the first cytokines induced from 6 hpi; while IL-1β expression showed a peak (11.3 times vs. the control) at 12 hpi and TNF-α expression had a plateau before an abrupt increase at 48 hpi reaching 6.3 times of the control at 72 hpi. Delayed but significant time-dependent expression started at 12 hpi and 48 hpi for CXCL10 and IL-6, respectively.

**Figure 5:**
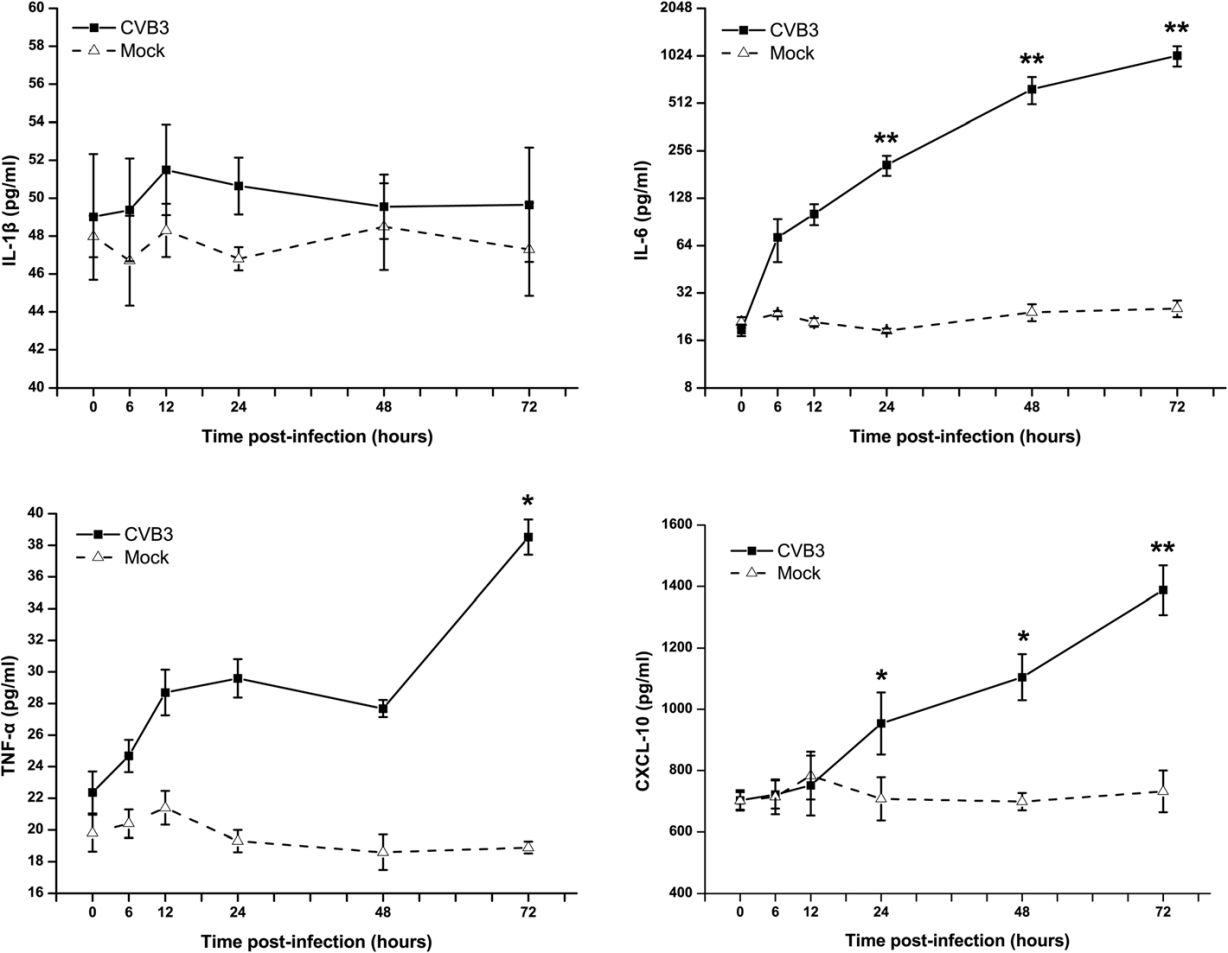
**Time course of cytokines production from astrocytes infected at an MOI of 2.** Levels of IL-1β, TNF-a, IL-6, and CXCL10 were determined at indicated time intervals. Values represent Mean ± SD from triplicate cultures from three independent experiments. * *p*<0.05 and **p<0.01 compared to mock infection.

The expression of four cytokines in the CVB3-infected astrocytes was detected in the culture supernatant. Figure 5 shows time course and magnitude of IL-1β, TNF-α, IL-6 and CXCL10 production. IL-1β and TNF-α induction occurred within 12 hpi, whereas IL-6 and CXCL10 levels gradually increased over 72 h.

## Discussion

Association of group B coxsackievirus (CVB) with the CNS infections have been previously shown by identification of antigens in or isolation of infectious particles of Coxsackieviruses from the brain tissues of patients with encephalitis [12,13]. CVB can also cause CNS complications in mice and neurons are reportedly susceptible to CVB in a mouse model [3]. However, no study has implicated major CNS immune cells astrocytes or microglia in the CVB pathogenesis. In this study, we report the CVB3 infectivity in murine primary astrocytes and the corresponding inflammatory response.

We used one- or two-day-old neonatal Balb/c mice because newborn pups are inherently more susceptible to coxsackievirus infection than are older mice and the susceptibility drops dramatically with age [3,14]. Cellular receptors are major determinants of viral pathogenesis, and thence a previous report that CAR expression level is significantly higher in the brain of newborn mice than adult mice [6] and the deduced amino acid sequence of a murine CAR homolog was 91% identical to that of the human CAR [15]. The presence of CAR in the murine astrocytes in this study suggests astrocytes could mediate CVB pathogenesis in the CNS. Besides, that CVB3 replicated in astrocytes and induced pronounced CPE (Figure 3) further suggests that CVB3 could cause direct damage to astrocytes.

Activated astrocytes are known to produce a wide variety of cytokines and chemokines [16,17]. Cytokine production in response to CVB3 has been described in other cell types, such as insulinoma cell line [18] and myocardial cells [19]. We observed upregulated mRNA expression levels in response to CVB3 infection of IL-1β, TNF-α, IL-6 and CXCL10 in astrocytes (Figure 4 and 5). Induced IL-1β mRNA expression was not correlated with the appearance of mature IL-1β in the supernatant, which could be due to impaired synthesis and liberation of IL-1β from infected cells and requires further research. Generally, cytokine release initiates a beneficial inflammatory and immune response although an exaggerated release is commonly detrimental and associated with adverse effects such as CNS disorders. IL-6 has been shown to be beneficial in the CNS because of its neurotrophic and neuroprotective effects. However, IL-6 and TNF-α production by astrocytes can lead to increased permeability of the BBB [20]. Many factors, particularly IL-1β and TNF-α, have been shown to induce IL-6 expression by astrocytes [21]. In consistent with this finding, TNF-α and IL-1β were stimulated before IL-6 release in our study. Marked increase in the expression of CXCL10 at both gene and protein level could be a part of innate immunity against CVB3. Previous studies have shown that the CXCL10 and its receptor CXCR3 play a vital role in the inflammatory response by attracting activated T lymphocytes to the CNS following viral infections such as Lymphocytic Choriomeningitis virus, Japanese encephalitis virus, and West Nile virus [22-24]. In addition to chemotaxis that contributes to neuropathology, CXCL10 has been reported to induce neuron apoptosis or direct damage in neuronal cells [25,26].

It is notable that CVB3 induced the death of infected astrocytes (Figure 3). As astrocytes are essential for providing trophic support to neurons and maintain synaptic functions, loss of astrocytes can induce significant neuronal dysfunction and damage. Certain cytokines such as TNF-α and CXCL10 are known to cause cell death directly [27,28]. In fact, when activated in an uncontrolled manner, astrocytes can release various substances, such as NO, superoxide anion, and inflammatory cytokines, triggering the cascade of events leading to neuron degeneration in the CNS [29]. Glial cells such as astrocytes and microglia cells, although are responsible for maintenance of brain integrity, may develop noxious effects upon their activation. The situation is exacerbated as the cytokines released from the infected astrocytes may induce secondary cytokine and inflammatory responses in the uninfected astrocytes, which could further promote cell death. Although we did not investigate full cytokine profile upon CVB3 infection in astrocytes, other direct treatment studies indicate that TNF-α triggers apoptosis in astrocytes [30], supporting the notion that CVB3-induced cell death could be related to the dysregulation of cytokines expression. It can be postulated that CVB3 infection lead to cell death via cytokine/chemokine-mediated pathways.

In conclusion, CVB3 is capable of infecting and replicating in primary murine astrocytes with resultant cytopathic effect and induction of pro-inflammatory cytokines (TNF-α, IL-1β, IL-6) and chemokine (CXCL10). Our findings suggest that i) CVB3 infection may contribute to encephalitis and/or meningitis via astrocyte destruction, and ii) permeability of the BBB and recruitment (not invasion) of inflammatory cells are a consequence of the cytokine/chemokine production.

## Materials and methods

### Animals

This study was approved by the Ethics Committee of Shantou University Medical College and conducted in conformity with the Experimental Animal Management Bill issued on November 14, 1988 (Decree No.2 of National Science and Technology Commission, China), and the National Institute of Health Guide for the Care and Use of Laboratory Animals (NIH Publications No.80-23, revised 1996). One- or two-day-old specific pathogen free Balb/c mice were purchased from Shantou University Medical College Laboratory Animal Center, Shantou, Guangdong, P.R. China.

### Virus enrichment and titration

Coxsackievirus B3 (Nancy strain) was kindly provided by Dr. Qihan Li (Institute of Medical Biology, Chinese Academy of Medical Science & Peking Union Medical College). CVB3 was propagated in Vero cells with Dulbecco’s Eagle’s minimum essential medium (DMEM) (Gibco, Life Technologies, USA) supplemented with 10% fetal bovine serum (FBS) (Gibco, Life Technologies, USA), Penicillin (20 U/ml) and Streptomycin (20 μg/ml). Monolayer of Vero cells was inoculated with the virus at a multiplicity of infection (MOI) of 0.1 and incubated at 37°C with 5% CO_2_ in a humidified incubator for 24 h. The viral culture supernatant was collected after centrifugation at 1500 *g* for 10 min and stored at −80°C until used.

Virus titer was determined based on the 50% tissue culture infectious dose (TCID_50_) as described previously [31]. Briefly, a 10-fold serial dilution of virus supernatant was added in duplicate to 80-90% confluent Vero cells in 96-well culture plates (Greiner Bio-One, FrickenHausen, Germany). The virus-infected cells were inspected daily for the appearance of typical Coxsackievirus-induced CPE using an inverted light microscope (IX750, Olympus, Japan). The TCID_50_ titer was calculated using Reed and Munch calculation method [32]. A batch of uninfected cells was used as control.

### Preparation of primary astrocytes and purity assay by immunofluorescence

Isolation of astrocytes from neonatal mice was performed as previously described [16]. Astrocytes were probed with Anti-Glial Fibrillary Acid Protein (GFAP) antibody. Nuclei were stained with Hoechst 33258 (1:1000, Beyotime, China). The purity of astrocytes was calculated by counting GFAP-positive cells in 10 random fluorescence fields under a Nikon 90i microscope.

### Detection of coxsackievirus receptor (CAR) and Glial Fibrillary Acid Protein (GFAP) on astrocytes by fluorescence staining

Monolayer astrocytes were grown on Poly-L-lysine coated glass cover slips. For fluorescence staining, cells were washed twice with phosphate buffered saline (PBS) and fixed in 4% paraformaldehyde. Cells were blocked and permeabilized with 0.1% Triton-X100/1% bovine serum albumin/PBS (PH 7.4) and incubated with Anti-Glial Fibrillary Acid Protein (GFAP) (1:500, eBioscience, CA, USA), followed by Alexa Fluor 488 goat anti-rabbit IgG (1:1000, Invitrogen, NY, USA) to identify astrocytes. The purity of astrocytes was determined by observing 10 random fluorescence fields.

To identify CAR expression on astrocytes, Anti-CAR (H-300, 1:200, Santa Cruz, CA, USA) was used as primary antibody, followed by Cy3 goat anti-mouse IgG (1:200, Beyotime, China). Nuclei were stained with Hoechst 33258 (1:1000, Beyotime, China) and examined with a Nikon 90i laser microscope.

### Virus infection of primary astrocytes

Astrocytes were cultured in a 12-well plate with DMEM-F12 supplemented with 10% FBS (10^5^ cells/well) at 37°C with 5% CO_2_ in a humidified incubator. We used MOI=0.1 for CVB3 replication assay and MOI=2 for CPE and cytokine assays, in which 99% of astrocytes were expected to get infected. At 90% confluence, the cultures were inoculated with CVB3. After adsorption for 1 h, the cultures were washed three times with PBS and replenished with fresh medium. At different post-infection time points, the supernatants were aliquoted and stored at −80°C until used for virus titration and analysis of cytokine production.

### Determination of astrocyte viability

Astrocytes were placed in a 96-well plate at density of 5000 cells/well. This density was chosen to ensure that cells are just confluent at the end of the assay. CVB3 was added to all wells at MOI of 2 except control wells. After adsorption for 1 h, the cultures were washed three times with PBS and replenished with fresh medium. The cell counting kit-8 (Dojindo, Kumamoto, Japan) was used to assay cell viability at different post-infection time points according to the manufacturer’s instructions.

### Quantification of mRNA by real time quantitative RT-PCR

Six batches of RNA were extracted from the infected astrocytes and control at 0 hpi, 6 hpi 12 hpi, 24 hpi, 48 hpi, and 72 hpi using Trizol reagent (Invitrogen Life Technologies). The RNA samples were used for cDNA synthesis with M-MLV Reverse Transcriptase (Invitrogen Life Technologies). Real-time quantitative PCR analysis of the expression level of IL-1β, TNF-α, IL-6, and CXCL10 was performed using the Platinum SYBR Green qPCR SuperMix-UDG (Invitrogen Life Technologies) and Applied Biosystems 7300 Real-Time PCR System (Applied Biosystems) following the manufacturers’ instructions. The primers used for real-time PCR analysis are listed in Table 1. The levels of gene expression were normalized against β-actin expression. For comparative purposes, estimation of the gene expressions for IL-1β, TNF-α, IL-6, and CXCL10 were determined according to the 2^-∆∆ CT^ method [33], where C_T_ is the cycle at which the detected signal is significantly above background signal. ∆∆C_T_ = [(C_T_ genes of post-infection− C_T_ β-actin) – (C_T_ gene of mock − C_T_ β-actin)], Fold change=2^-∆∆CT^. Quantification was performed in triplicate using cDNAs from three independent experiments.

**Table 1:** Primers used in real-time PCR

**Gene name**	**Forward / Reverse primer sequences**	**GenBank acc. no.**
IL-1β	5′-GATGTCCAACTTCACCTTCA-3′	NM_008361.3
	5′-ACAAACTTCTGCCTGACGA-3′	
IL-6	5′-GCCTTCTTGGGACTGATGCTGGT-3′	NM_031168
	5′-GGTCTTGGTCCTTAGCCACTCCT	
TNF-α	5′-TCAACCTCCTCTCTGCCGTCAAG-3′	M13049
	5′-TCCAGGTCACTGTCCCAGCATCT-3′	
CXCL-10	5′-GTGCTGCCGTCATTTTCTGCCTC-3′	NM_021274
	5′-GGGTAAAGGGGAGTGATGGAGAG-3′	
β-actin	5′-GCAGCTCCTTCGTTGCCGGT-3′	NM_007393.3
	5′-GGGGCCACACGCAGCTCATT-3′	

### Measurement of cytokines and chemokine production by ELISA

Levels of IL-1β, TNF-α, IL-6, and CXCL10 were assessed using ELISA kits: IL-1β (Cat.No. BM6002), TNF-α (Cat.No. BM607/2), and IL-6 (Cat.No. BM603/2) were purchased from eBioscience. CXCL10 was purchased from Bender Medsystems (Cat.No. BM6018), ELISA assay was performed according to manufacturer’s instructions. Plates were read using SPECTRA max 340PC (Molecular Devices, USA) at 450 nm wavelength. To inactivate virus infectivity before assay, the supernatants of infected and uninfected culture were irradiated with UV light for 30 min. Ultraviolet irradiation had no effect on theses cytokines concentration (data not shown).

### Statistical analysis

The data are presented as mean ± SD. The significance of difference between values was estimated by means of one-way analysis of variance (ANOVA) with Fisher’s LSD post-hoc test. A *p*-value of less than 0.05 was considered statistically significant. The statistical analyses were done using the SPSS 13.0 for windows (Chicago, USA).

## Competing interests

The authors declare that they have no competing interests.

## Authors’ contributions

Conceived and designed the experiments: KSL, JZ, GFW. Performed the experiments: JZ, GFW, WZL, DGZ, XXC, GX, ZWJ. Analyzed the data: JZ, GFW, XXC. Wrote the paper: JZ, KSL. All authors read and approved the final manuscript.
